# Advances on Greener Asymmetric Synthesis of Antiviral Drugs via Organocatalysis

**DOI:** 10.3390/ph14111125

**Published:** 2021-11-04

**Authors:** Everton M. da Silva, Hérika D. A. Vidal, Arlene G. Corrêa

**Affiliations:** Centre of Excellence for Research on Sustainable Chemistry, Department of Chemistry, Federal University of São Carlos, São Carlos 13565-905, SP, Brazil; machado.everton93@gmail.com (E.M.d.S.); herikavidal2@gmail.com (H.D.A.V.)

**Keywords:** antivirals, green chemistry, selectivity, asymmetric synthesis, organocatalysis

## Abstract

Viral infections cause many severe human diseases, being responsible for remarkably high mortality rates. In this sense, both the academy and the pharmaceutical industry are continuously searching for new compounds with antiviral activity, and in addition, face the challenge of developing greener and more efficient methods to synthesize these compounds. This becomes even more important with drugs possessing stereogenic centers as highly enantioselective processes are required. In this minireview, the advances achieved to improve synthetic routes efficiency and sustainability of important commercially antiviral chiral drugs are discussed, highlighting the use of organocatalytic methods.

## 1. Introduction

The increasing prevalence of diverse microbial infections, as well as the emergence and re-emergence of viral epidemics with high morbidity and mortality rates, are a significant public health threat. Despite the continuous production of antiviral drugs and vaccines in the global market, viruses remain one of the leading causes of deadly human diseases. Effective control of viral diseases, caused particularly by zika, dengue, herpes simplex, human immunodeficiency (HIV), and Ebola virus, remain promising goals amidst the mutating viral strains [1].

It is estimated that an average of 3 million deaths per year in the last decades worldwide were caused by different viral pathogens, in particular hepatitis, AIDS, and influenza [2]. More recently, the coronavirus disease (COVID-19) caused by SARS-CoV-2 is creating tremendous human suffering, and, to date, no effective drug is available to treat the disease directly. According to the World Health Organization, globally, between January 2020 and September 2021, there have been more than 234 million confirmed cases of COVID-19 and 4.8 million deaths [3].

In this sense, both the academy and the pharmaceutical industry continuously search for new compounds with antiviral activity [4]. Emphasis should focus on orally available drugs for outpatient use, if possible, and on identifying combination therapies that combat viral and immune-mediated pathologies, extend the effectiveness of therapeutic windows, and reduce drug resistance. As the emergence of viral diseases is expected to accelerate, proactive programs to develop broadly active family-specific and cross-family antiviral therapeutics will be critical to prepare for future disease [5,6].

Chiral molecules have played a prominent role in developing new drugs, as individual stereoisomers may exhibit marked differences in pharmacodynamic, pharmacokinetic, and toxicological properties [7]. Therefore, there is considerable interest in fully characterizing and examining both enantiomers in the early stages of new drug development [8]. In this regard, the pharmaceutical industries either exploit the chiral switching practice from already marketed racemates or develop de novo enantiomerically pure compounds [9].

One crucial step in therapeutics is the synthesis of drug candidates under development, their critical intermediates and the active pharmaceutical ingredients (APIs) since it encompasses the search for routes with high efficacy, good yields, and less impact in the environment [10]. Furthermore, the synthesis of APIs requires large-scale analysis of the process; thus, using fewer toxic reagents, greener solvents, more efficient methods, and waste reduction is essential for the sustainability of the production [11].

The three pillars of asymmetric catalysis are biocatalysis, metal catalysis, and organocatalysis [12]. The biological catalysts, such as isolated enzymes or whole cells, are increasingly used on an industrial scale and are particularly favored for hydrolytic reactions [13]. The rapid development of organometallic chemistry and catalysis has made numerous breakthrough contributions to organic synthesis; however, such widespread use of metal compounds has highlighted the significance of environmental and toxicity issues [14]. In this respect, a recent trend in the chemical science and pharmaceutical industry is the dismissal of catalytic metal complexes and their replacement with organocatalysts. The efficacy and synthetic versatility of asymmetric organocatalysis [15,16] and the use of a plethora of highly effective small-molecule organocatalysts have enriched the field of organic synthesis, including chiral proline derivatives, *N*-heterocyclic carbenes, chiral thioureas, and Brønsted acids as well as phase-transfer catalysts (PTC), such as the quaternary ammonium salts derived from cinchona alkaloids [17].

Moreover, in the context of green and sustainable syntheses, continuous flow chemistry offers exceptional opportunities to accelerate, integrate, simplify, scale up, and automatize chemical reactions. Early this year, Ötvös and Kappe reviewed the flow chemistry-based approaches for the synthesis of chiral APIs and their advanced stereogenic intermediates, covering the utilization of biocatalysis, organometallic catalysis, and organocatalysis [18].

A significant number of antiviral agents used in clinical practice are amino acids, short peptides, or peptidomimetics, and among them are several HIV protease inhibitors (e.g., lopinavir, atazanavir) [19], HCV protease inhibitors (e.g., grazoprevir, glecaprevir) [20], and HCV NS5A protein inhibitors (e.g., daclatasvir, pibrentasvir) [21]. Recently, Skwarecki et al. [22] reviewed the synthesis of these antiviral agents with particular attention to the synthetic aspects of non-proteinogenic amino acid components.

In Table 1, we present selected chiral antiviral drugs, the anti-HIV agents being the major examples [23], highlighting the asymmetric strategy employed as a crucial step to achieve their total synthesis. It is worth noting that most of the synthetic routes use chiral pools, including amino acids, as starting materials (entries 3, 4, 6, 12, 13, 15–17). Chiral resolution using either enzymes or metal catalysts has also been successfully employed (entries 1, 2, 8, 9, 14) and only in a few cases have enantioselective organocatalyzed methods been employed (entries 10 and 11).

This minireview discusses the advances achieved to improve synthetic routes efficiency and sustainability of important commercially antiviral chiral drugs. The organocatalytic methods were selected to be presented in more detail, and the proposed mechanism pathways for introducing the stereogenic centers are highlighted.

## 2. Synthesis of Efavirenz

Efavirenz (**1**) is a reverse transcriptase inhibitor drug used to treat infections caused by the human immunodeficiency virus (HIV) type I. Approved in 1998 by the FDA, it has been used in combination with other drugs for the treatment of AIDS [54]. 

**Scheme 1:**
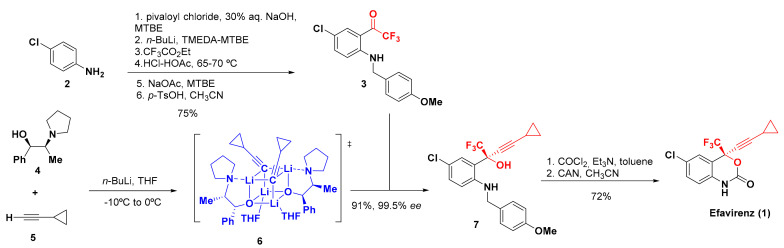
First total synthesis of efavirenz (**1**).

Pierce et al. [55] reported in 1998 an enantioselective synthesis of efavirenz in nine steps with 49% overall yield. The key step involves an enantioselective addition of the cyclopropylacetylide 5 to trifluoromethyl ketone 3 in the presence of the additive (1*R*,2*S*)-*N*-pyrrolidinylnorephedrine (**4**) (Scheme 1). Initially, 4-chloroaniline (**2**) was transformed into ketoaniline 3 with a 75% yield over six steps. The enantioselective alkynylation occurred with the formation of the chiral complex, obtained by the reaction of a mixture of chiral ligand 4 and cyclopropyl acetylene (**5**) with *n*-butyllithium at −10 to 0 °C in a toluene–hexane mixture (THF). Then it was cooled below −50 °C, and the ketoaniline was added. The enantioselectivity of this transformation is dependent on the *N*-protecting group of compound **3**. The mixture of acetylide degenerated at low temperature exists as a complex mixture of species. A stable aggregate has been fully characterized as the 2:2 symmetric cubic tetramer, and it was observed that optimal reaction conversion and enantioselectivity require strict control of reagent stoichiometry and reaction conditions. The amino alcohol 7 was obtained with a 91% yield and 95.5% *ee*, then was converted into benzoxazinone, and the deprotection occurred by ceric ammonium nitrate (CAN), giving efavirenz (**1**) in 72% yield. This route is only suitable for small-scale synthesis since an equal amount of *p*-methoxy benzaldehyde is formed but not efficiently removed upon crystallization. Furthermore, a significant quantity of cerium salt was generated as a waste, raising concerns for the scale up of chemical reactions. Although the chiral ligand 4 was employed in an equimolar amount, it could be recovered in 98% yield and recycled.

**Scheme 2:**
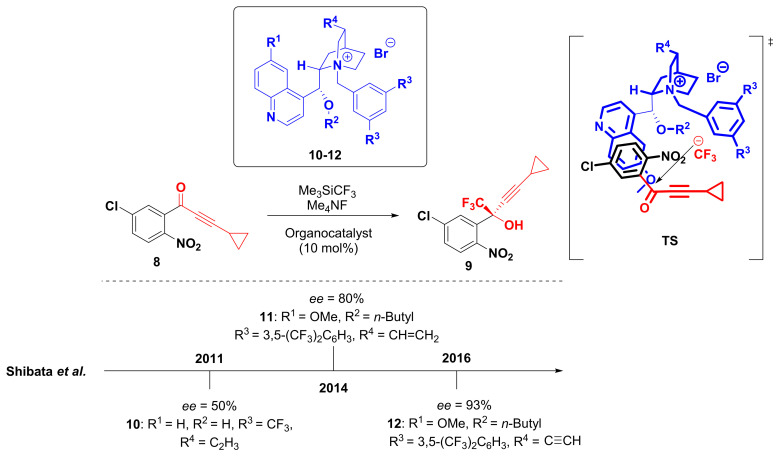
Organocatalyzed trifluoromethylation of alkynyl ketone 8.

In 2011, Shibata et al. [56] reported the first synthesis of efavirenz based on a metal-free, enantioselective trifluoromethylation of alkynyl ketone (**8**) with trimethyl(trifluoromethyl)-silane (Me_3_SiCF_3_). This transformation is different from the previously reported methods because an organocatalytic based on a cinchonidine derivative 10 was used, however, with only 50% *ee*. The enantioselectivity was improved (up to 80% *ee*) in 2014 by the same group [57], using a quinine-based catalyst 11 bearing a butoxy group (Scheme 2). Nevertheless, the organocatalytic process was still less efficient than the organometallic enantioselectivity approach. Thus, in 2016, Shibata et al. [58] reported using a quaternary ammonium salt of cinchona alkaloids having an alkynyl group (**12**). Structurally, the best catalyst used has only the different ethynyl groups, so the 3D conformation of the transition state (TS) is similar to the others. However, the catalyst has a closed structure between the ethynyl group and the quinoline ring, and the alkenyl ketones can be stabilized by a π−π interaction with the quinoline ring and the methoxy group help in positioning the ketones by a steric interaction. Some modifications in the conditions of this reaction were necessary, but the trifluoromethylation using a novel catalyst occurred with 88% yield and 93% *ee*. With the trifluoro methylated compound in hand, the synthesis of efavirenz was completed in two steps. Reduction of the nitro group to aniline was obtained in 83% yield and 91% *ee*. The aniline was treated with 4-nitrophenyl chloroformate in the presence of KHCO_3_ to obtain efavirenz (**1**) in 89% yield. Using *n*-hexane/CH_2_Cl_2_ by single recrystallization, the enantiopurity was increased to 99%.

## 3. Synthesis of Oseltamivir

Tamiflu, the phosphate of (−)-oseltamivir (**13**), is the most efficient drug for the treatment and prevention of flu. The mechanism of action fundamentally involves the inhibition of the neuraminidase enzyme of type A and type B viruses [59].

**Scheme 3:**
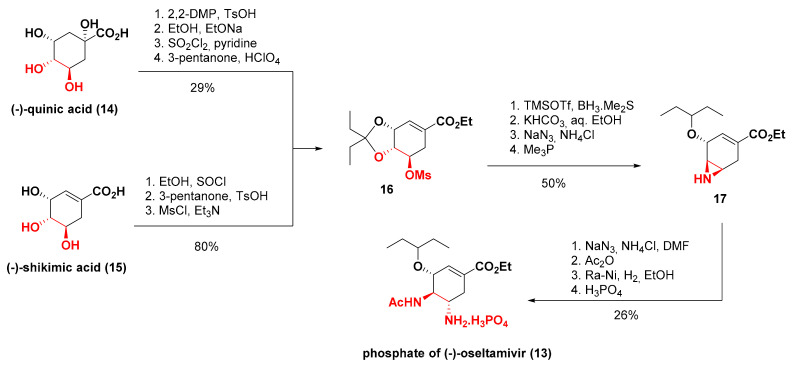
First total synthesis of phosphate of (−)-oseltamivir (**13**).

The first total synthesis of the phosphate of (−)-oseltamivir was described in 1998 by Rohloff et al. (Scheme 3) [60]. The synthesis is based on two natural products, (−)-quinic acid (**14**) and (−)-shikimic acid (**15**), which makes it challenging to be employed on an industrial scale quickly and inexpensively [61]. The two routes pass for the same intermediate, the 3,4-pentyline ketal 16. In the route of the quinic acid (**14**), the 3,4-pentyline ketal 16 was formed in four steps, in 29% yield, and in the route of (−)-shikimic acid (**15**), the 3,4-pentyline ketal 16 was obtained in three steps in 80% yield. The 3,4-pentyline ketal 16 was transformed in pentyl ether and converted into an epoxide converted in the azido alcohol, and finally in the aziridine 17 with 50% yield over four steps. The aziridine was converted in the azido amine that was directly acylated. Finally, the azide was reduced to amine and the salt of oseltamivir. H_3_PO_4_ (**13**) was isolated after four steps with a 26% overall yield. The final product was obtained in 11 steps and 10.4% yield from (−)-shikimic acid (**15**) and in 12 steps in 3.7% yield from (−)-quinic acid (**14**).

**Scheme 4:**
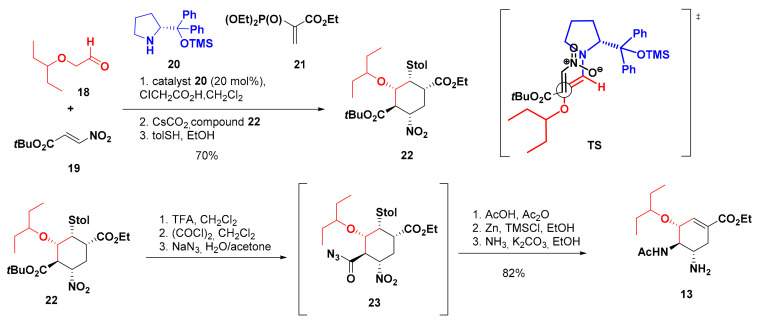
Asymmetric organocatalyzed synthesis of oseltamivir (**13**).

The syntheses based on the natural products presented low yields, in addition to numerous reaction steps that required several purification processes. With this in mind, in 2009, Ishikawa and et al. [62] reported a high-yielding synthesis of oseltamivir via three one-pot operations. The chiral cyclohexane was built based on organocatalyzed reaction, and the process minimized chemical waste generation and time of purifications. Diphenylprolinol silyl ether (**20**) was an effective organocatalyst, promoting an asymmetric reaction with excellent enantioselectivities. In the first step, the organocatalyst provides the Michael adduct with alkoxyaldehyde (**18**) and nitroalkane (**19**) in a quantitative yield and 96% *ee* (Scheme 4). Then, a Michael reaction between the nitroalkane and vinyl phosphonate 21 occurs, and the formed phosphonate undergoes an intramolecular Horner–Wadsworth–Emmons reaction with the formyl group to generate ethyl cyclohexenecarboxylate. Treatment of the mixture with *p*-toluenethiol in the presence of Cs_2_CO_3_ gives the product 22 in 70% yield. With compound **22** ready, the deprotection occurs, followed by the conversion of carboxylic acid in an acyl azide 23 in three steps. This azide is submitted to a domino Curtius rearrangement, followed by reduction of the NO_2_ to the amine. A retro-Michael reaction of the thiol afforded oseltamivir, which was obtained in 82% yield from compound **22** after purification by acid—base extraction. In summary, the efficiency of the Hayashi—Jørgensen organocatalyst was described in the total enantioselective synthesis of (−)-oseltamivir that was carried out in 9 reactions, in three one-pot operations, and just one purification by chromatographic column with 57% overall yield.

**Scheme 5:**
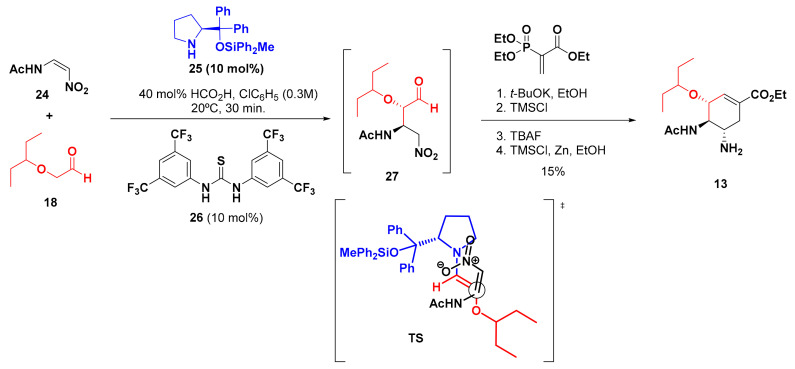
Time economy synthesis of oseltamivir (**13**).

Although this route was very efficient, the reaction time was quite long. Thus, based on previous reports published in the literature [62,63], Hayashi and Ogasawara described in 2016 a time economical total synthesis of (−)-oseltamivir in 60 min (Scheme 5) [64]. In the first step, the Michael reaction of nitroalkene 24 and α-alkoxyaldehyde 18 proceeds in the presence of diphenylprolinol silyl ether 25 and Schreiner’s thiourea 26, affording product 27 that was not isolated. The organocatalyst was vital for generating enamine, and the thiourea activated the nitroalkene via hydrogen bonding. The cyclohexene was formed via a domino Michael and Horner−Wadsworth−Emmons reactions. Then, after protonation with TMSCl, epimerization using TBAF and reduction of the nitro group in the presence of Zn, the desired compound **13** was obtained after purification by column chromatography in 15% yield, via a one-pot procedure. In summary, the total synthesis of oseltamivir was described in a single reactor over five steps, being the route optimized not only in yield and selectivity but also in reaction time.

## 4. Synthesis of Zanamivir

Zanamivir (**28**) is used in the treatment of influenza type A and B and was approved in the United States in 1999 [65]. Zanamivir inactivates the influenza viral neuraminidase, an enzyme responsible for cleaving sialic acid residues in newly formed viruses. Such inhibition results in virus aggregation on the host cell surface, limiting the extent of infection and accelerating recovery from the disease [66].

**Scheme 6:**
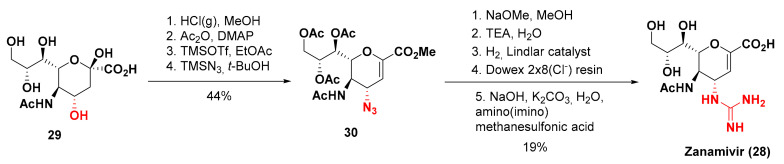
First scalable synthesis of zanamivir.

The first scalable synthesis of zanamivir was described by Chandler et al. [67]. The route started with acetylneuraminic acid (**29**), which was converted into a methyl ester, and the hydroxy groups were acetylated (Scheme 6). Using TMSOTf, the oxazoline was formed and selectively converted in the azide 30. The acetate protecting group was removed by a catalytic amount of NaOMe to gain water solubility, following hydrolysis of the methyl ester with aqueous TEA to give the triethylammonium salt. This salt was hydrogenated in the presence of Lindlar catalyst to give the amine. Then, in addition to the aminoiminomethanesulfonic acid with the amino acid, the guanidine functionality was introduced. The crystalline zanamivir (**28**) was isolated by ion-exchange chromatography. Several synthetic steps were likely to be performed on a gram scale. Most of the intermediates were isolated by recrystallization, however, in one case, desalting and freeze-drying are also necessary, and the use of the ion-exchange chromatography to obtain the pure product. The overall yield of the 9-step synthesis was only 8.3%.

**Scheme 7:**
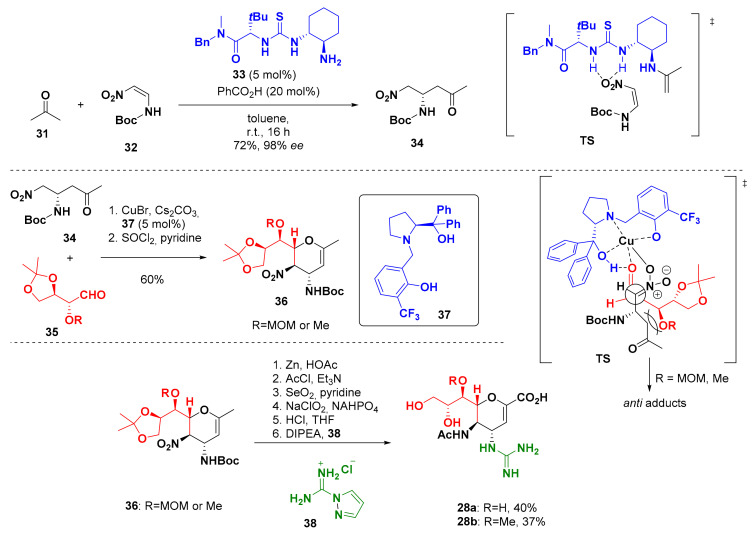
Total synthesis of zanamivir starting from D-araboascorbic acid.

Due to the high cost of sialic acid, and considering the need for production on a global scale, in 2014, Tian et al. [68] developed a total synthesis of zanamivir in 13 linear steps, starting from *D*-araboascorbic acid (Scheme 7). Initially, the (*Z*)-*tert*-butyl (2-nitro vinyl)carbamate (**34**) was prepared in three steps from nitromethane with 72% yield. The organocatalyzed Michael addition of compound **32** in acetone was investigated, and the best conditions were achieved using thiourea catalyst 33 in toluene and benzoic acid as an additive. The reaction produced compound **34** in 72% yield and 98% *ee* after recrystallization of the crude product from ethyl acetate/petroleum ether (1:10).

With compound **34** ready, the Henry reaction with aldehyde 35 was investigated. The aldehyde 35 was synthesized from *D*-araboascorbic acid [69]. Based on previous reports for anti-selective addition, a combination of CuBr_2_ and the proline-derived catalyst 37 was used, and after spontaneous cyclization and subsequent dehydration with thionyl chloride and pyridine, selective formation of the anti-product 36 was observed with 60% yield, together with the formation of the undesired product in 8% yield. By observing the possible transition states, the volume of the protective group interferes with the reaction, which proceeds favorably by the proposed transition state, due to the repulsion between the protective group and the carbamate side chain (the only formation of the non-selective product was observed when the TBS was used). Therefore, smaller protection groups (MOM and Me) were used, thus favoring the formation of the anti-selective product. Then, the hydroxyl protecting groups of 36 were changed to obtain the carboxylic acid via oxidation of the methyl group. The protecting groups were all removed with HCl and finally, after guanidination with 38, led to the formation of zanamivir with single recrystallization. The overall yield was 18%, and the synthetic route offered advantages due to the cost of the starting materials, being an inexpensive and accessible alternative for the manufacture of this anti-influenza drug.

## 5. Synthesis of Letermovir

Letermovir (**39**) is a drug approved by the FDA in 2017 for the treatment of infections caused by the human cytomegalovirus (HCMV) [70]. Severe, acute HCMV infections most commonly involve symptomatic infection of the gastrointestinal tract, liver, and central nervous system and hematological manifestations of the eye, lung, or arterial or venous thrombosis. The viral terminase complex is particular to herpesviruses since no cellular protein carrying out its function has been identified in mammalian cells, making this viral complex a good target for antivirals. Letermovir targets pUL56 of the HCMV terminase complex [71].

**Scheme 8:**
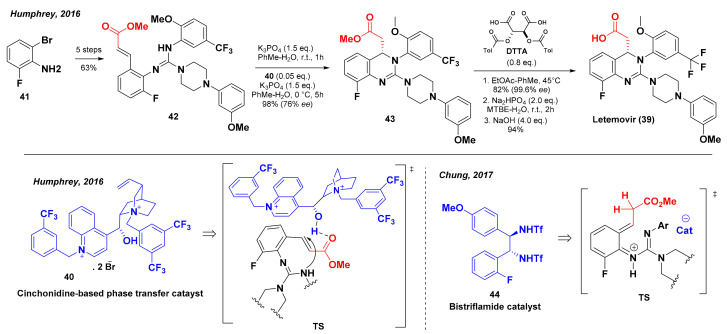
Synthesis of letermovir (**39**) using organocatalysts 40 or 44.

Humphrey et al. [72] proposed an asymmetric synthesis for obtaining 39 with high enantioselectivity (Scheme 8). The total synthesis of letermovir (**39**) was carried out in eight steps from commercially available starting materials, and the key step was an aza-Michael reaction catalyzed by a cinchonidine-based phase transfer catalyst (PTC) 40, producing about 1t of product. The authors suggest some factors that interfere in enantioselectivities, such as stirring rate, concentration, and equivalents of K_3_PO_4_ aqueous solution, and the counterions Br^−^ and PO_4_^3−^ show low results. In terms of Green Chemistry, the method shows an excellent enantiomeric excess (99.6% *ee*) using a cinchonidine-based organocatalytic under mild conditions with an overall yield > 60% in eight steps, providing letermovir (**39**) on a large scale. Based on previous work [73], the TS was proposed by theoretical studies showing that the use of the catalyst promotes stereodiscrimination (*S*) in the ring closure as a result of the interaction between catalyst/substrate, hydrogen bonding (O-H), substrate conformation, π-stacking interactions, and interactions with the benzyl-CF_3_ group of the catalyst.

Furthermore, the same group reported the use of chiral bistriflamide 44 as an organocatalyst to promote the enantioselective cyclization for the synthesis of letermovir (**39**). According to the mechanism pathway, supported by density functional theory (DFT) calculations and kinetics studies, the bistriflamide 44 acts as a Brønsted acid (Scheme 8). Initially, using 42 occurs the protonation of nitrogen by bistriflamide 44, followed by tautomerization and cyclization to produce guanidine 43. The catalyst promotes a double donation of hydrogens, so the TS is formed by the tautomerization of the starting material. This method showed high yields (96%) and a good enantiomeric ratio (84:16 *er*), being applied on a large scale for the synthesis of letermovir (**39**), demonstrating efficiency with reduced drawbacks when compared with the PTC-catalyzed method.

## 6. Synthesis of Ruxolitinib

Ruxolitinib (**45**) was the first drug used to treat myelofibrosis and polycythemia vera (PCV), approved by the FDA in 2011. Ruxolitinib acts specifically in inhibiting the protein tyrosine janus kinases (JAK) 1 and 2, promoting a decrease in inflammation and an inhibition of cellular proliferation [74].

**Scheme 9:**
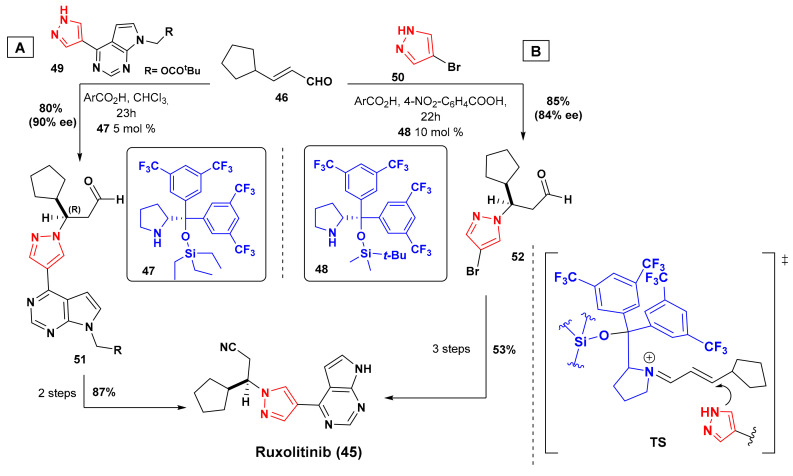
Synthesis of ruxolitinib (**45**) using a proline-derived organocatalyst.

One of the key structural elements of this drug is the chiral pyrazole ring, which defines stereochemistry. Lin et al. [75] developed the synthesis of ruxolitinib via aza-Michael addition, based on studies by Jørgensen [76] with proline-derived organocatalysts. According to the proposed mechanism, the addition by the *Re* face is favored with 94% *ee*, while the *Si* face is protected by the catalyst. This methodology presents two possibilities, both using cyclopentanecarbaldehyde (**46**) as starting material. In the first route (Scheme 9A), the aldehyde 46 reacted with compound **49** catalyzed by 47, furnishing *R*-51 in 80% yield and 90% *ee*. In the next two steps, 45 is obtained. In the second synthetic route (Scheme 9B), compound **46** reacted with 4-bromo-1H-pyrazole (**50**) catalyzed by 48, and the product *R*-52 is obtained with 85% yield and 84% *ee*. The following steps are the conversion of the aldehyde in nitrile, and 45 is obtained with 53% yields. In both routes, the TS was proposed based on Jørgensen’s study where an addition on the *Re* face is favored, so in reactions using the more steric hindered organocatalysts, higher enantioselectivities were observed. The authors do not present information on either the recovery of the catalysts or the performance tests on large scales, although the presented methodologies have good prospects in green chemistry through the use of organocatalysts and an efficient asymmetric route.

**Scheme 10:**
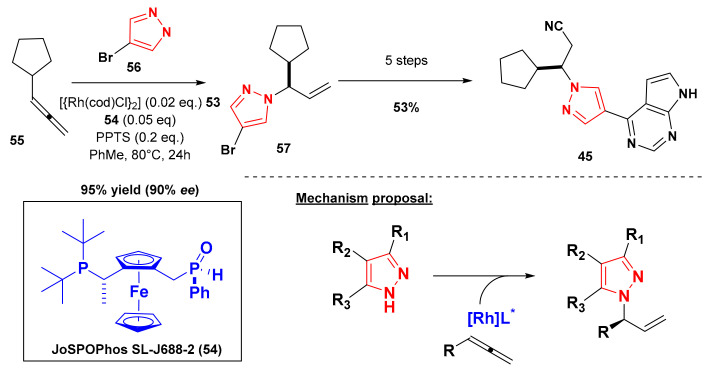
Rhodium-catalyzed asymmetric synthesis of ruxolitinib (**45**).

Hayld et al. describe the synthesis of ruxolitinib (**45**) [77] based on strategies developed by their group on the enantioselectivity of pyrazoles using a rhodium/JoSPOPhos (**53** and **54**) catalyst system (Scheme 10) with a high atom economy. Initially, with 4-bromopyrazole (56) and cyclopentylalene (**55**) in the rhodium-catalyzed coupling, it was possible to obtain the product 57 in the gram scale, with 95% yield and 90% *ee*. Then, after five steps, 45 is obtained with a 53% overall yield. The authors do not present detailed information about the mechanism, it is only shown that upon tautomerism of the pyrazole ring and the use of 54, it is possible to obtain only the desired product and that the use of pyridinium *p*-toluenesulfonate (PPTS) plays a fundamental role in selectivity.

## 7. Synthesis of Remdesivir

Remdesivir (**58**), a prodrug of the parent nucleoside GS441524 [78], was initially developed for the treatment of Ebola; however, due to the pandemic caused by SARS-CoV-2, after clinical tests, it was approved in 2020 by the FDA to be used in severe cases of the COVID-19 [79]. The active form of remdesivir functions as a nucleoside analog and inhibits coronaviruses’ RNA-dependent RNA polymerase (RdRp) [80].

**Scheme 11:**
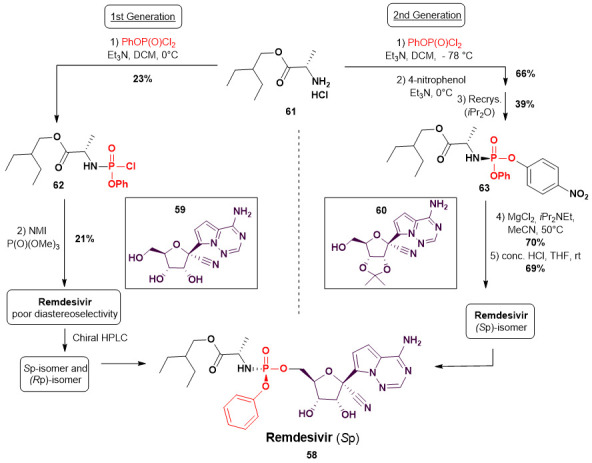
First- and second-generation synthesis of remdesivir (**58**).

The main challenge in synthesizing remdesivir is establishing the stereogenic center in the phosphorus atom [81,82]. The first- and second-generation syntheses of remdesivir (**58**) are shown in Scheme 11. In both synthetic routes, the same starting material 61 is employed, which is submitted to phosphorylation with the formation of compounds **62** and **63**, respectively. After the coupling with nucleobases (**59** or **60**), in the first generation, the two isomers formed are separated using chiral HPLC [83]. In the second-generation synthesis occurs a selective nucleophilic displacement, where there is no racemic production after phosphorylation, so only the *S* enantiomer is obtained upon recrystallization, using hydrochloric acid. Although it is possible to obtain the enantioenriched product 58, the phosphorylation occurs with the lowest yield, so improvements in this step of the synthesis were needed.

**Scheme 12:**
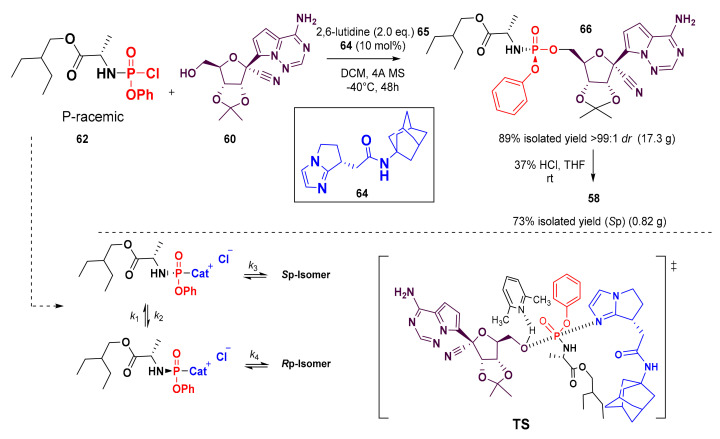
Synthesis of remdesivir (**58**) using the imidazole organocatalyst 64.

Wang et al. [84] developed a synthesis of remdesivir based on the use of imidazole organocatalysts. A kinetic study was carried out evaluating the velocity constants (k) (Scheme 12), where it was observed that the key point of using the catalyst is that it can promote the k1/k2 racemization and that it is enantioselective in the formation of product 58. The first test used compounds **62** and **60** without any catalyst, observing only traces of the product with a diastereoisomeric ratio (*dr*) of 1.1:1 (*S*p:*R*p). Achiral catalysts were used, and the *N*-methyl imidazole (NMI) presented the best result with 1.5:1 *dr*. Then, the chiral bicyclic imidazole catalysts, modified with benzyl, acetyl ester, *tert*-butyl carbonate, and carbamates were used, and the catalyst 64, modified with adamantinyl group, showed the best results with 11.2:1 *dr*. In addition, 2,6-lutidine (**65**) was used as a base at low temperature (−40 °C), resulting in an increase of the diastereoselectivity to 21.8:1. The catalyst concentration did not interfere in the enantioselectivity, and in cases of using a lower concentration, a longer reaction time is necessary.

The mechanistic proposal initially suggested a transition state involving a simultaneous action of phosphoryl chloride and nucleoside by the catalyst. Theoretical studies were carried out and showed that the *S*p configuration favored 3.5 kcal. mol, per the experimental data. Finally, they explored this method in large-scale reaction (10 g), employing 10 mol% catalysts 64 at 40 °C, and the products were obtained with 21.2:1 *dr*, the *S*p enantiomer was obtained with 89% yield and > 99:1 *dr* (85% yield by recrystallization and 4% yield of residual mother liquor by preparative HPLC). The authors emphasize the atom economy of the method and synthetic efficiency. Moreover, the catalyst was recovered without any damage and could be reused in subsequent cycles. The high yield obtained in the gram-scale reaction shows its potential application in the industry.

**Scheme 13:**
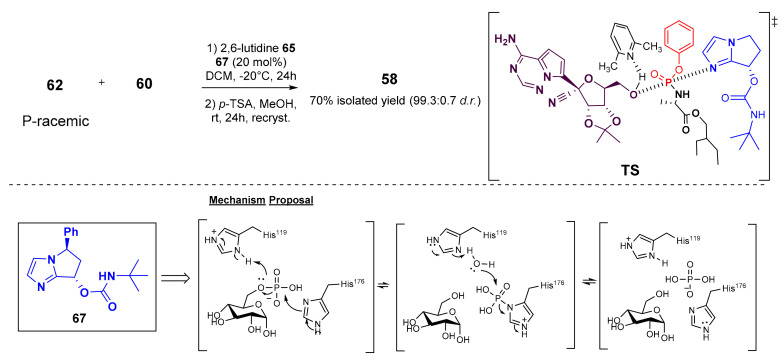
One-pot synthesis of remdesivir (**58**) using the imidazole-derived catalyst 67.

In 2021, Gannedi et al. [85] studied a strategy for obtaining the stereogenic center of the phosphorus atom via a one-pot synthesis also employing an imidazole-derived catalyst (Scheme 13). Several bicyclic chiral imidazole derivatives were tested, but catalyst 67 with phenyl group showed the best result, with 25:1 *dr* and 97% yield. After identifying catalyst 67 as the most effective, further optimization was carried out in reaction time, temperature, and catalyst loading. The authors concluded that −20 °C for 24 h was ideal since the increase in reaction time caused a decrease in catalytic activity. Equally, the reduction in temperature to −40 °C promoted a discrepant increase in selectivity, and with a decrease in temperature to −78 °C, no reaction was observed. Concerning catalyst 67, the decrease in percentage promoted a decrease in selectivity, and extrapolation in the amount of 100% molar catalyst promoted 42:1 *dr*; however, to avoid further purification of diester, the catalyst loading of 20 mol% was maintained. Several tests to increase the reaction scale were carried out. In the 1g scale, after the first step with the substrates 62 and 60, catalyst 67, *p*-TSA, and methanol were added in the same flask reaction (one-pot) at room temperature. This resulted in the removal of the isopropylidene group and presented a mixture of 58 with its diastereoisomer, which was purified by recrystallization with a 73% yield and 99.6:0.6 *dr*. On the 10 g scale, a 70% yield and 99.3:0.7 *dr* of the desired product were obtained, and the catalyst was recovered in an 83% yield.

The reaction pathway was inspired by the mechanism of histidine-dependent glucose 6-hydrolysis catalyzed by glucose 6-phosphate phosphatase (Scheme 13). It can be seen that the imidazole residues of His119 and His176 may act as a proton donor and receptor. Based on this finding, catalyst 67 was proposed with a similar chemical structure. Furthermore, it was observed that the catalyst configuration directly influences the product stereochemistry, that is, a catalyst with (*S*)-configuration favored the (*S*)-product. The proposed transition state was based on Wang’s study [83], which uses similar imidazole organocatalysts.

## 8. Conclusions

Viral infections cause many serious human diseases, being responsible for remarkably high mortality rates. In this sense, both the academy and the pharmaceutical industry are continuously searching for new compounds with antiviral activity, and in addition, face the challenge of developing greener and more efficient methods to synthesize these compounds. In this minireview, the developments in the asymmetric synthesis of antivirals based on green methods were summarized. The approaches using organocatalysts in the key steps were emphasized, including the proposed mechanism to these enantioselective transformations. Although the yield and enantioselectivity of these reactions are generally good to excellent, the catalyst loading is still high (5–10 mol%), and recovery and recycling require tedious steps of purification. Moreover, one-pot reactions, which reduce the generation of waste from purification steps and the continuous flow synthesis, are paving the way for a greener scale up of chemical reactions. These methods are especially important for developing countries, where it is essential to enable local drug manufacturing and improve access to medicines.

## Figures and Tables

**Table 1:** Selected chiral antiviral drugs.

Entry	API	Chemical Structure	Treatment or Indications and Mode of Action	Enantioselective Synthesis: Chiral Pool or Key Step
1	Amprenavir	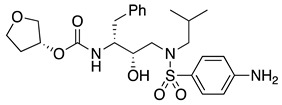	AIDS, HIV protease inhibitor	(a) Co(salen) catalyzed kinetic resolution of 2-(1-azido-2-phenylethyl)oxirane [24] (b) from *L*-acid malic and *L*-phenylalanine [25]
2	Abacavir	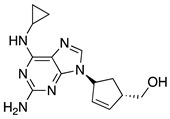	AIDS and hepatitis B virus (HBV), reverse transcriptase inhibitor	(a) chiral resolution using γ-lactamase [26] (b) Oppolzer-sultam as auxiliary then asymmetric rhodium-catalyzed coupling using Josiphos 003-1 [27]
3	Cobicistat	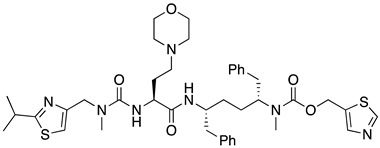	AIDS, human CYP3A proteins inhibitor	chiral amino acids as starting materials [28,29]
4	Daclatasvir	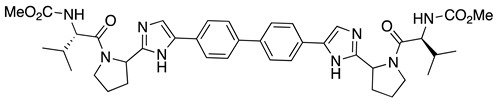	chronic hepatitis C virus (HCV) genotype 3, inhibitor of the NS5A	continuous-flow synthesis with chiral amino acids as starting materials [30]
5	Dolutegravir	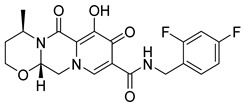	AIDS, integrase strand transfer inhibitor	(*R*)-3-amino-1-butanol as starting material [31]
6	Elvitegravir	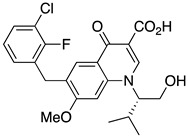	AIDS, HIV integrase inhibitor	(*S*)-valinol as starting material [32]
7	Emtricitabine	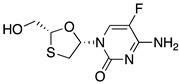	AIDS and HBV, reverse transcriptase inhibitor	*L*-menthol as chiral auxiliary [33]
8	Indinavir	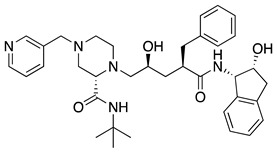	AIDS, HIV-1 protease inhibitor	(a) (*R*)-glycidol as starting material [34] (b) from indanone via enantioselective ester hydrolysis with *Rhizopus oryzae* [35] (c) (1*S*,2*R*)-*cis*-aminoindanol as starting material [36]
9	Lamivudine	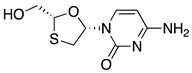	AIDS and HBV, reverse transcriptase inhibitor	(a) enzymatic dynamic kinetic resolution [37] (b) (*S*)-lactic acid as starting material [38] (c) *L*-menthol as chiral auxiliary [39]
10	Maraviroc	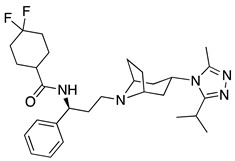	AIDS, antagonist of the CCR5 receptor	(a) (*S*)-*tert*-butanesulfinamide as chiral auxiliary [40] (b) aza-Michael/hemiacetal reaction using the Hayashi–Jørgensen organocatalyst [41]
11	Nelfinavir	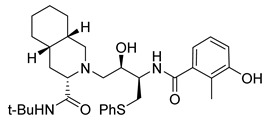	AIDS, HIV protease inhibitor	(a) Ti catalyzed asymmetric aminolysis of epoxide [42] (b) starting from chiral sulfoxide [43] (c) bromocyclization of bisallylic catalyzed by (*S*)-BINAP [44]
12	Ritonavir	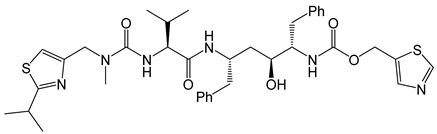	AIDS, HIV protease inhibitor	chiral amino acids as starting materials [45,46]
13	Saquinavir	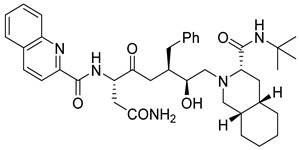	AIDS, HIV protease inhibitor	chiral amino acids as starting materials [47]
14	Simeprevir	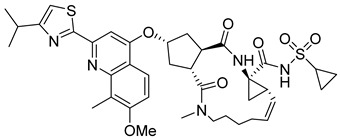	HCV, NS3/4A protease inhibitor	(a) chiral resolution using pig liver esterase [48] (b) chiral resolution using cinchonidine [49]
15	Telaprevir	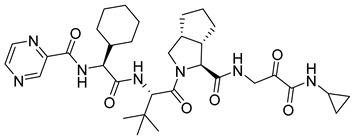	HCV, NS3/4A protease inhibitor	lipase mediated desymmetrization of 1,2-cyclopentanedimethanol and chiral amino acids [50]
16	Tenofoviralafenamide	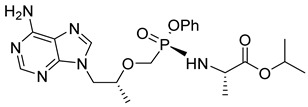	AIDS and HBV, nucleotide reverse transcriptase inhibitor	(*R*)-9-[2-(phosphonomethoxy) propyl] adenine (PMPA) [51] from *L*-threonine and *L*-alanine as starting materials [52]
17	Valacyclovir	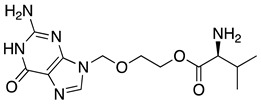	Herpes, prodrug converted to acyclovir, a DNA polymerase inhibitor	from *L*-valine [53]

## Data Availability

Data sharing is not applicable. No new data were created or analyzed in this study.
